# Challenges and Opportunities for Therapeutic Targeting of Calmodulin Kinase II in Heart

**DOI:** 10.3389/fphar.2020.00035

**Published:** 2020-02-05

**Authors:** Drew Nassal, Daniel Gratz, Thomas J. Hund

**Affiliations:** ^1^The Frick Center for Heart Failure and Arrhythmia and Dorothy M. Davis Heart and Lung Research Institute, The Ohio State University Wexner Medical Center, Columbus, OH, United States; ^2^Department of Biomedical Engineering, College of Engineering, The Ohio State University, Columbus, OH, United States; ^3^Department of Internal Medicine, The Ohio State University Wexner Medical Center, Columbus, OH, United States

**Keywords:** calmodulin kinase II, arrhythmias, heart failure, cardiovascular pharmacology, cardiac remodeling

## Abstract

Heart failure remains a major health burden around the world. Despite great progress in delineation of molecular mechanisms underlying development of disease, standard therapy has not advanced at the same pace. The multifunctional signaling molecule Ca^2+^/calmodulin-dependent protein kinase II (CaMKII) has received considerable attention over recent years for its central role in maladaptive remodeling and arrhythmias in the setting of chronic disease. However, these basic science discoveries have yet to translate into new therapies for human patients. This review addresses both the promise and barriers to developing translational therapies that target CaMKII signaling to abrogate pathologic remodeling in the setting of chronic disease. Efforts in small molecule design are discussed, as well as alternative targeting approaches that exploit novel avenues for compound delivery and/or genetic approaches to affect cardiac CaMKII signaling. These alternative strategies provide hope for overcoming some of the challenges that have limited the development of new therapies.

## Introduction

For the past 50 years, therapy based on antagonism of beta adrenergic receptors, angiotensin converting enzyme (ACE), and/or AT2 receptors has been the staple for heart failure (HF) treatment (Bers, 2005). However, the incidence of HF and associated mortality rates continue to grow at an alarming pace, highlighting the need for novel therapies to target pathways that drive HF progression. While the field has shown tremendous strides in understanding mechanisms associated with pathologic remodeling leading to HF, translation of this information into new and effective therapies has been less successful. This review discusses the multifunctional signaling molecule Ca^2+^/calmodulin-dependent protein kinase II (CaMKII) as an example of both the promise and challenges of developing translational therapies based on our evolving understanding of pathologic remodeling in the setting of chronic disease. Despite the wealth of basic research supporting CaMKII as a master regulator of pathways important for cardiac remodeling and arrhythmia, the field has yet to witness translation of these findings to the clinic. The gap between advances at the bench and new treatments in human patients has not gone unnoticed, and efforts are underway that will hopefully fix the pipeline for development of effective therapy. In this review, we address a brief history of progress in small molecule drug design, followed by consideration of alternative approaches that take advantage of recent advances in novel delivery mechanisms as well as genetic approaches for manipulating CaMKII signaling in the heart. These alternative strategies address the ability to circumvent the challenges and limitations of small molecule drug design while highlighting next-generation therapeutic paradigms.

## CaMKII Expression, Structure, and Function in Heart

CaMKII is a serine/threonine kinase important for translating changes in the levels of intracellular Ca^2+^/calmodulin (and other critical second messengers) into adaptations in cell function through direct phosphorylation of a large number of target proteins (Swaminathan et al., 2012; Westenbrink et al., 2013). Diversity in metazoan CaMKII expression arises from existence of four isoforms, α, β, γ, and δ, each encoded by a distinct gene. While α and β isoforms are primarily expressed in the brain where they regulate indispensable mechanisms of long-term potentiation/depression necessary for learning and memory (Silva et al., 1992a; Silva et al., 1992b; Stevens et al., 1994; Coultrap et al., 2014), the δ and γ isoforms are broadly expressed in multiple tissues, including the heart (Bennett et al., 1983; Tobimatsu and Fujisawa, 1989). Notably, CaMKIIδ, the predominant cardiac isoform, undergoes alternative splicing to give rise to CaMKIIδ_b_ and CaMKIIδ_c_ variants (Edman and Schulman, 1994). This splicing event confers CaMKIIδ_b_ with a nuclear localization signal not expressed in CaMKIIδ_c_ leading to differential subcellular distribution (Srinivasan et al., 1994; Ramirez et al., 1997). Despite important differences, there is high degree of homology across CaMKII isoforms, posing significant challenges for therapy around CaMKII inhibition. Given the ubiquitous nature of CaMKII with expression throughout the body, it is no small task to effectively target isoforms/splice variants involved in cardiac pathophysiology while avoiding those important for normal physiology in heart and other organ systems (e.g., brain) (Little et al., 2009; Peng et al., 2010; Lu et al., 2011b; Beckendorf et al., 2018).

**Figure 1:**
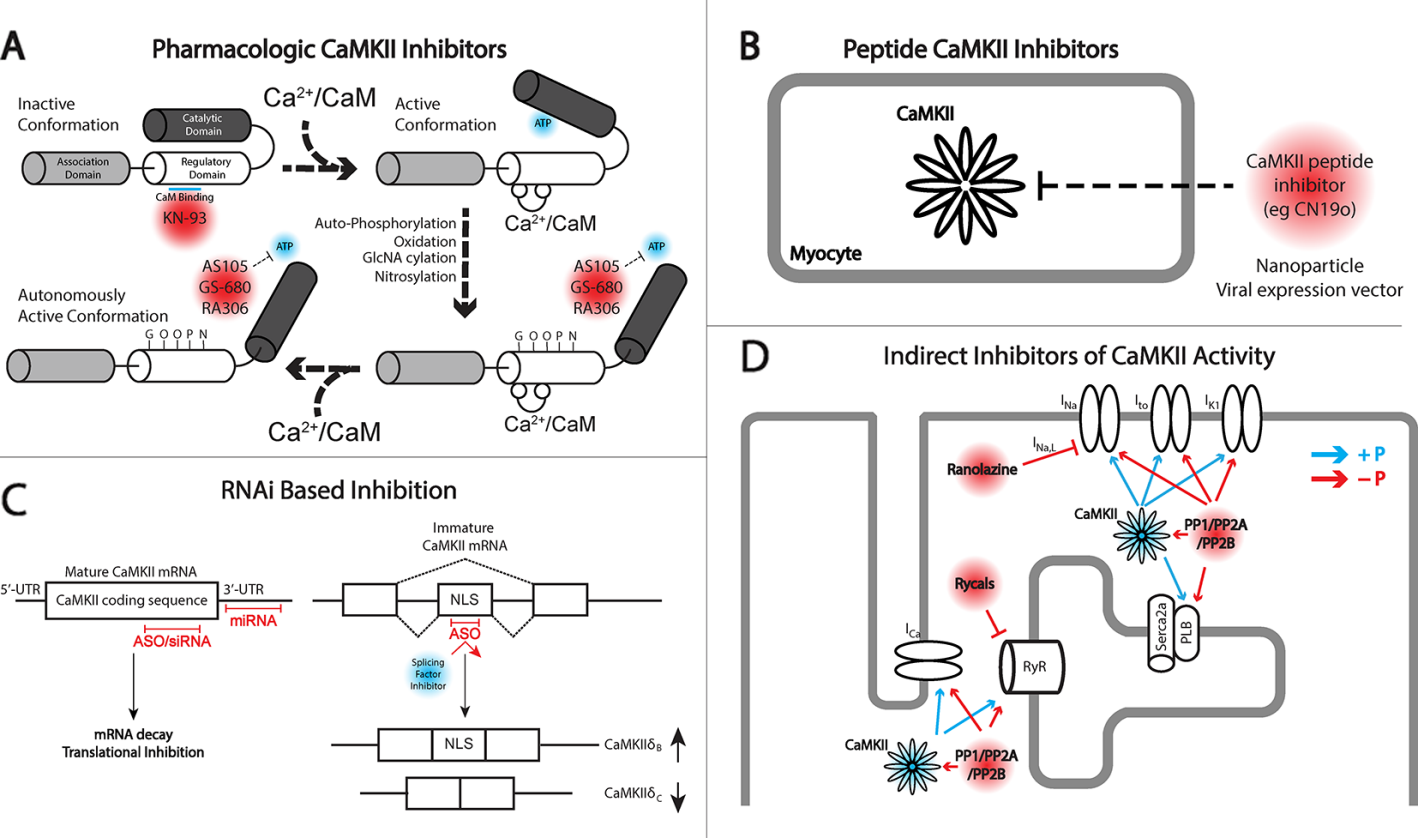
Different approaches for targeting Ca^2+^/calmodulin-dependent protein kinase II (CaMKII) signaling. **(A)** Depiction of inactive and active monomers of CaMKII showing the association, regulatory, and catalytic domains. The association domain is responsible for interaction with other CaMKII monomers and is necessary for forming the holoenzyme structure, consisting of 12 monomers. In its inactive state, the catalytic domain is obscured by interaction with the regulatory domain. This interaction is disrupted upon the binding of Ca^2+^/CaM leading to autophosphorylation by neighboring CaMKII monomers, in addition to other posttranslational modifications including oxidation, GlcNAcylation, and nitrosylation, maintaining CaMKII activation even upon release of Ca^2+^/CaM. KN-93 is a known allosteric inhibitor of CaM binding and therefore preferentially targets CaMKII in the inactive state. CaMKII inhibitors AS105, GS-680, and RA306 are novel pyrimidine–based, ATP-competitive inhibitors that inhibit the activated catalytic domain of CaMKII and represent potential therapeutic agents for translational CaMKII inhibition. **(B)** Peptide inhibitors of CaMKII (e.g., CN19o, refined from CaMKIItide) show favorable selectivity and potency for CaMKII inhibition but face challenges in delivery and bioavailability. Both viral gene delivery and novel advances in nanoparticles offer opportunities for delivery of these agents to the heart. **(C)** RNA interference (RNAi) is a novel approach for inhibiting CaMKII activity at the transcript level. Antisense oligonucleotides (ASOs), small interfering RNA (siRNA), and miRNAs provide opportunity for degrading CaMKII transcripts and/or inhibiting protein translation. ASOs can also be used to interfere with recruitment of splicing factors to enhance concentrations of CaMKIIδ_B_ which has been shown to have cardioprotective effects. **(D)** Indirect inhibition of CaMKII can be achieved by regulating downstream targets of CaMKII kinase activity. An example comes from the late Na^+^ current (I_Na,L_) inhibitor ranolazine, or the RyR stabilizing agents, rycals. Alternatively, protein phosphatases may be targeted to antagonize kinase activity in cardiomyocytes. The development of phosphatase activators for cancer therapeutics may offer opportunity for drug applications in cardiovascular disease.

Before considering strategies for targeting CaMKII, it is important to briefly discuss the kinase structure/function relationship. A single molecule of CaMKII is composed of three domains: (1) the N-terminal catalytic domain responsible for ATP binding and kinase function; (2) the regulatory domain responsible for binding Ca^2+^/calmodulin and subsequent kinase activation; and (3) the C-terminal association domain responsible for the oligomerization of individual CaMKII molecules to create a mature dodecameric-holoenzyme (**Figure 1A**). As will be discussed, the N-terminal catalytic domain has received the most attention for translatable drug design. Inactive CaMKII is folded in a closed conformation where the regulatory domain of each CaMKII monomer acts as a substrate binding the catalytic domain. Additionally, adjacent regulatory domains within the dodecameric structure block the binding of target substrates and ATP, maintaining a self-inhibited state. Activation of CaMKII occurs when Ca^2+^/calmodulin binds to a defined region in the regulatory domain resulting in a conformation shift that releases the catalytic domain, exposing the kinase substrate and ATP binding sites. Importantly, the regulatory domains of neighboring CaMKII monomers within the holoenzyme are themselves substrates for active CaMKII kinase activity, specifically at Thr287. Phosphorylation of this residue dramatically increases affinity for Ca^2+^/calmodulin and also prevents reassociation of the regulatory and catalytic domains, creating sustained CaMKII activity through beat-to-beat fluctuations in Ca^2+^ cycling.

In addition to the autophosphorylation at Thr287 maintaining the active conformation, other posttranslational modifications have been identified on the regulatory domain, which maintain an active state and have been associated with cardiac disease states. These include the oxidation of Met281/282 in response to elevated reactive oxygen species (Erickson et al., 2008; He et al., 2011; Purohit et al., 2013; Anderson, 2015), O-linked N-acetylglucosamine (GlcNAcylation) targeting Ser279 as a result of elevated glucose levels (Erickson et al., 2008; Erickson et al., 2013), and reports of S-nitrosylation predicted on Cys290 as a result of increased NO production upon β-AR stimulation (Gutierrez et al., 2013; Erickson et al., 2015). Together, these posttranslational modifications establish a diverse set of pathways that contribute to sustained activation of CaMKII, reinforcing the strong association between CaMKII activity and the development of cardiac disease.

The physiologic targets and consequences of cardiac CaMKII signaling have been thoroughly addressed in previous review articles (Anderson et al., 2011; Westenbrink et al., 2013; Mattiazzi et al., 2015; Mustroph et al., 2017). Therefore, we will only briefly discuss CaMKII targets in the context of cardiac remodeling and disease to lay the foundation for more detailed treatment of efforts to inhibit CaMKII. Ample experimental evidence causally links chronic CaMKII activity to development of cardiac disease and arrhythmias (Maier and Bers, 2002; Swaminathan et al., 2012; Westenbrink et al., 2013). Animal models have shown proof-of-concept studies that transgenic overexpression of CaMKII is sufficient to induce structural and electrical remodeling in the heart, leading to compromised contractility and increased risk for sudden cardiac death (Zhang et al., 2002; Zhang et al., 2003; Wagner et al., 2011). Likewise, genetic and chemical inhibition of CaMKII has been shown to confer protection from the development of dilated cardiomyopathy and sustained contractile performance, following both pressure overload and ischemic stress (Zhang et al., 2005; Backs et al., 2009; Ling et al., 2009). Important when considering translation, human HF has also been associated with an increased expression/activity of CaMKII (Hoch et al., 1999; Kirchhefer et al., 1999). The central role for CaMKII in development of disease stems from its regulation of proteins involved in critical cell functions from Ca^2+^ cycling to mitochondrial function. CaMKII has been implicated in pathologic phosphorylation of a number of Ca^2+^ handling proteins including phospholamban, leading to activation of the sarcoplasmic reticulum (SR) ATP-driven Ca^2+^ pump SERCA2a (Mattiazzi and Kranias, 2014); the ryanodine receptor SR Ca^2+^ release channel (RyR2) (Witcher et al., 1991; Lokuta et al., 1995; Wehrens, 2011), promoting increased channel open probability and SR Ca^2+^ leak; and the L-type Ca^2+^ channel Ca_v_1.2 and associated β-subunits, potentiating current amplitude and slowing inactivation (Hudmon et al., 2005; Grueter et al., 2006). Collectively, these events not only promote activation of hypertrophic remodeling cascades but also heighten the risk for inappropriate membrane potential depolarizations (afterdepolarizations) that serve as arrhythmia triggers (Wu et al., 2002). CaMKII-dependent phosphorylation of other ion channels like the primary cardiac voltage-gated Na^+^ channel Na_v_1.5 enhances late depolarizing current, leading to prolonged action potentials, further disrupting Ca^2+^ handling and providing additional substrates for the formation of arrhythmogenic afterdepolarizations (Koval et al., 2012; Glynn et al., 2015; Howard et al., 2018). Mitochondrial Ca^2+^ entry has also been identified as a target for CaMKII with consequences for mitochondrial function and apoptosis (Joiner et al., 2012; Erickson et al., 2013).

Beyond ion channels, CaMKII has been found to target chromatin remodeling protein class 2 histone deacetylases 4 and 5 (HDAC4/5). The phosphorylation of these proteins induces their nuclear export, freeing the transcription factor MEF2 from its repressed state, contributing to hypertrophic remodeling (Backs et al., 2006; Wu et al., 2006; Backs et al., 2008). Relevant to pathology, CaMKII signaling is notable for the presence of unique dynamical properties including 1) feedback where CaMKII affects downstream targets that in turn alter an input signal for CaMKII activation (Yao et al., 2011; Morotti et al., 2014; Onal et al., 2017); and 2) bistability where CaMKII is capable of toggling between resting and activated equilibrium states (Michalski, 2013). Together, these findings point to CaMKII as a prime candidate for next-generation therapies in managing heart disease in human patients. Despite our thorough understanding of CaMKII structure and function, the development of therapeutics based on this knowledge is in its infancy. Our goal for the remainder of the review will be to provide a comprehensive look at the progressing therapeutic opportunities for inhibiting CaMKII for HF management. Recent reviews have excellently covered the history and progress of CaMKII inhibition (Pellicena and Schulman, 2014; Mustroph et al., 2017). Here, we will provide an update of recent efforts toward the development of translational pharmacologic agents while considering more novel and unconsidered opportunities in drug delivery and genetic approaches (summarized in **Table 1**).

**Table 1:** Properties and limitations of Ca^2+^/calmodulin-dependent protein kinase II (CaMKII) inhibitory agents.

Inhibitor	Class	Mode of action	Properties and limitations
AS105	Pharmacologic	ATP competitive	Half maximal inhibitory concentration IC50 in the low nM range; shown to improve Ca^2+^ handling in isolated mouse and human cardiomyocytes. Limitations: Activity against CaMKIIα/β/γ, other off-targets, and bioavailability for *in vivo* delivery unknown.
GS-680	Pharmacologic	ATP competitive	IC50 of 2.3 nM for CaMKIIδ with weaker selectivity against CaMKIIα/β/γ and weak interaction with human ether-a-go-go-related gene (hERG). Shown to restore contractility and Ca^2+^ handling in human trabeculae from failing hearts. Limitations: Bioavailability and *in vivo* testing unknown and evidence of potential negative ionotropic effect reversed by isoproterenol treatment.
RA306	Pharmacologic	ATP competitive	IC50 in the 10 nM range for CaMKIIδ/γ with weaker potency against CaMKIIα/β and relatively weak inhibition against hERG, K_v_4.3, Na_v_1.5, and Ca_v_1.2. *In vivo* oral delivery restored contractility in genetic mouse model of dilated cardiomyopathy with minimal drug delivery to the brain. Limitations: Potential inhibition of other kinases associated with cardiac remodeling. Effects on electrical remodeling are not defined. Additional detail needed on acquired disease states like pressure overload.
AC3I/AIP	Peptide	Substrate competitor	IC50 of ~3 µM (AC3I) and 40 nM (AIP). Cardiac specific transgenic models shown to effectively attenuate hypertrophic remodeling, heart failure (HF), and arrhythmias. Limitations: Comprehensive screening of off-targets would be necessary for translational approaches. Existing screens show specificity for CaMKII; however, all isoforms are targeted with equal potency, mandating cardiac specific expression. Bioavailability and cell permeation nonexistent without use of viral vector delivery or potential use of novel nanoparticle delivery.
CaMKIIN	Peptide	Substrate/regulatory domain competitor	IC50 of 50 nM; however, refinement of the core peptide sequence to the most recent generation (CN19o) has enhanced specificity to CaMKII and improved the IC50 to 0.4 nM. Membrane and mitochondrial associated transgenic expression in mice reduced inflammatory signaling and mitochondrial stress following ischemic injury. Limitations: May impair CaMKII interaction with scaffolding proteins leading to disruption of kinase signaling domains. Lack of bioavailability and cell permeation, requiring viral vector or novel nanoparticle delivery.
Small interfering RNA (siRNA)/antisense oligonucleotide (ASO)/miRNA	RNAi	Degradation of mRNA, translational inhibition, or alternative splicing	Genetic knockout (KO) of CaMKII in mouse lines has led to improved cardiac performance in multiple disease models. Limitations: *in vivo* therapeutic delivery of RNAi-based agents has not been tested for translational application. Additional limitations include CaMKII targeting in unintended tissues from system delivery.
Ranolazine	Indirect inhibitor of CaMKII signaling	Inhibits late *I*_Na_	Shown to reduce late *I_Na_* and prevent hypertrophy, HF, and arrhythmias in animal models. Limitations: Clinical trials show uncertain impact preventing atrial fibrillation recurrence, ventricular tachycardia (VT)/ventricular fibrillation (VF), or improving functional cardiac output in hypertrophic cardiomyopathy.
Rycals (JTV519, S107)	Indirect inhibitor of CaMKII signaling	Stabilizes RyR2	Shown to improve Ca^2+^ handling and ventricular function while protecting against arrhythmias and HF development in both rodents and large animal studies. Clinical investigations performed with S107 to target RyR1 expressed in skeletal muscle for muscular dystrophy treatment
Phosphatase activators	Indirect inhibitor of CaMKII signaling	Dephosphorylation of CaMKII substrates	PP2A activator FTY720 has shown protective capability. Current trend toward phosphatase activators in cancer therapeutics may provide opportunity to examine cardiac effects. Limitations: Transgenic overexpression of phosphatase subunits has been associated with cardiac disease.

## Pharmacologic Inhibitors of CaMKII

The approach of using pharmacologic inhibitors to target CaMKII activity has been used extensively in basic research with less progress in translational medicine, which is somewhat surprising given the widespread use of protein kinase inhibitors in cancer therapeutics for targeting tumor proliferation and cell survival (Bhullar et al., 2018). In fact, protein kinases are the second most targeted group of proteins, currently with 37 kinase inhibitors having received Food and Drug Administration (FDA) approval for cancer treatment, with another 150 in clinical trials (Bhullar et al., 2018). However, similar compounds have not been successfully developed for therapeutic purposes in the cardiac field due in part to a higher threshold for safety requirements, historical investment being more directed at ion channel blockers for anti-arrhythmics, and a larger burden of clinical trial costs and more uncertain return on investment compared to other therapeutics (Fordyce et al., 2015). Given the growing awareness of the slowdown in cardiovascular drug development (Fordyce et al., 2015) and the dominant academic literature on the contribution of CaMKII in pathologic cardiac remodeling, it seems the community is open and motivated for new pharmacologic drugs for CaMKII inhibition (**Figure 1A**).

Among the first small molecule inhibitors developed for CaMKII was KN-93 (Sumi et al., 1991), which has become one of the most widely used inhibitors in basic research. While its design was not intended for translational use, its prolific use over the years and unique mode of CaMKII inhibition make it important to discuss. While most kinase inhibitors target the ATP-binding domain, KN-93 is unique in that it allosterically disrupts CaM binding and stabilizes the interaction between the enzymatic and regulatory domains as evidenced by measured FRET signals associated with inactive versus activated structures of CaMKII (Erickson et al., 2011; Erickson et al., 2013). However, this mode of inhibition is thought to be less effective at inhibiting already active CaMKII, including autonomously activated CaMKII as a result of Thr287 autophosphorylation (Vest et al., 2010), which could also extend to additional autonomous modifications of oxidation and GlcNacylation. Another important limitation of KN-93 is that it is not particularly specific or potent against CaMKIIδ, with a half maximal inhibitory concentration (IC50) in a range of 1–4 µM. While KN-93 is reasonably selective for CaMKII over other protein kinases (Gao et al., 2013), it has been found to have a range of off-target effects, including voltage-gated potassium channels (Ledoux et al., 1999; Rezazadeh et al., 2006; Hegyi et al., 2015), L-type Ca^2+^ channels (Anderson et al., 1998), inositol triphosphate receptor Ca^2+^ release (Smyth et al., 2002), and even calmodulin (Johnson et al., 2019).

While KN-93 has proven a valuable research tool, it has not been until recently that concerted efforts have been made to move pharmacological CaMKII inhibition to the clinic. An early effort from the now defunct Scios sought to characterize CaMKII/activity relationships to identify new strategies for inhibiting CaMKII (Mavunkel et al., 2008). From this work, they developed the compound Scios 15b, a cell-permeable, ATP-competitive, pyridimine-based molecule that inhibits CaMKII with an IC50 of 9 nM *in vitro* and 320 nM *in situ*. These initial cell-based assays evaluated CaMKII inhibition by the extent to which it impaired CaMKII-dependent phosphorylation of vimentin. While the compound was not directly investigated with respect to its impact on pathological cardiac remodeling, this work laid the groundwork for the next generation of CaMKII pharmacologics that are being developed, particularly the pyrimidine, ATP-competitive compounds.

Indeed, a more recent novel CaMKII inhibitor, AS105, was also designed as a pyrimidine-based, ATP-competitive molecule by Allosteros Therapeutics following computational optimization of pyrimidine-based CaMKIIδ inhibitors (Neef et al., 2018). Achieving an IC50 in the low nanomolar range for *in vitro* binding, this drug was shown to therapeutically inhibit CaMKII in isolated cardiomyocytes from CaMKIIδ_C_-overexpressing mice with HF by reducing SR Ca^2+^ leak, improving SR Ca^2+^ loading and Ca^2+^ transient amplitude, and restoring myocyte fractional shortening. Importantly, it was shown to not negatively impact basal excitation–contraction coupling in cardiomyocytes. Moreover, it was able to reduce SR Ca^2+^ leak within isolated human atrial myocytes and suppress arrhythmogenic Ca^2+^ release events. These studies provide promise for the development of a novel CaMKII inhibitor; however, it remains to be seen how specific this drug is for CaMKIIδ compared to CaMKIIα/β/γ and other potential off-targets, as well as its bioavailability.

Another pyrimidine-based, ATP-competitive molecule, GS-680, has been developed by Gilead Sciences (Lebek et al., 2018). GS-680 has a reported CaMKIIδ biochemical IC50 of 2.3 nM, with values 3.1-, 8.7-, and 22.5-fold less potent for CaMKIIɣ, α, and β, suggesting a selective potency of CaMKII inhibition for isotypes expressed within the myocardium, minimizing the risk of neuronal side effects. Measurement of phosphorylation levels of phospholamban in neonatal rat ventricular myocytes and neurons treated with GS-680 yielded estimates of EC50 of 98.9 nM and 9,005 nM, respectively, for CaMKII inhibition, consistent with selectivity of the compound for cardiac CaMKII. Moreover, assessment of additional safety factors found the drug to have an EC50 of 3,000 nM for human ether-a-go-go-related gene (hERG) channel inhibition, minimizing the risk of QT prolongation. GS-680 was shown to effectively inhibit premature atrial contractions (PACs) in human atrial trabeculae by exposure to increased external Ca^2+^ (3.5 mM) and isoproterenol (100 nM) and did so in a dose-dependent manner, completely eliminating PACs at 100 and 300 nM concentrations, which occurred in 50% of nontreated samples. In parallel, GS-680 was found to reduce Ca^2+^ sparks and SR Ca^2+^ leak and attenuated triggered activity, as well as increased action potential amplitude and maximal upstroke velocity within isolated human atrial myocytes. However, the drug was found to impair systolic atrial contractions, which was negated after pretreating the tissue with isoproterenol, suggesting that CaMKII inhibition at baseline had a negative ionotropic impact that could be compensated by PKA stimulation. Consistent with this idea, human HF ventricular trabeculae preparations, which are characterized by increased PKA activity and negative force–frequency relationships, experienced improved contractility and increased Ca^2+^ transients at higher pacing frequencies when treated with GS-680, showing that this compound holds promise for the potential treatment of both atrial arrhythmias and HF ventricular remodeling.

Like the compounds just discussed, the most recently designed compound named RA306 developed by Sanofi R&D was derived from a chemical optimization program based on the pyrimidine ATP-competitive class of CaMKII inhibitors (Beauverger et al., 2019). The drug was found to be a potent inhibitor of human CaMKIIδ and ɣ, the two main cardiac isoforms, having an IC50 of 15 and 25 nM, respectively, in enzymatic caliper assays and below 3 nM for each isoform in a more sensitive P33 assay. CaMKIIβ was also inhibited with an IC50 of 61 nM, and CaMKIIα was weakly inhibited with an IC50 of 420 nM (assessed using caliper assays). There were a limited number of other protein kinases found to be inhibited by the drug including MLK1, SIK, and Pyk2, which have also been associated with adverse cardiac remodeling. RA306 also displayed a favorable ion channel selectivity profile, showing IC50 values for hERG, K_v_4.3, and Na_v_1.5 over 30 and 14 µM for Ca_v_1.2. The therapeutic potential of this compound was tested in a transgenic mouse model of dilated cardiomyopathy, but unique to these studies was the delivery of the drug as an orally bioavailable agent, giving it significant advantage for potential clinical investigation. The drug was found to improve ejection fraction in a genetic mouse model of dilated cardiomyopathy, which associated with reduced levels of phosphorylated phospholamban (Thr17), a direct target of CaMKIIδ. Critically, a five-fold lower exposure for the drug was found in the brain compared to the heart, limiting the potential for off-target impacts of CaMKII inhibition in the brain.

The last several years has seen a strong push in the development of translational pharmacologic agents to inhibit CaMKII for the treatment of pathologic heart conditions. While only one compound (RA306) has been shown to be orally bioavailable, several new agents are available with favorable selectivity and potency profiles. Therefore, based on these efforts, it seems reasonable to remain optimistic about the development of a translationally relevant and novel HF pharmacologic.

## Peptide Inhibitors of CaMKII

Peptide inhibitors (**Figure 1B**) represent some of the most effective agents to modulate CaMKII for research purposes. The identification of the auto-inhibitory state of the regulatory domain fueled the development of a long inhibitory peptide lacking both the CaM binding domain (Payne et al., 1988; Malinow et al., 1989) and the autophosphorylation residue Thr287 (mutated to be nonphosphorylatable). The result was the generation of the peptide inhibitors autocamtide-2-related inhibitory peptide (AIP) (Ishida et al., 1995) and autocamtide-3 derived inhibitory peptide (AC3-I) (Braun and Schulman, 1995), able to bind the CaMKII catalytic domain but unable to be displaced by canonical CaMKII activation mechanisms. As such, these inhibitors have been delivered through pharmacologic and genetic means to robustly inhibit CaMKII pathologic signaling *in vitro* and *in vivo*, mitigating HF, increasing cardiac function, and preventing lethal cardiac arrhythmias (Zhang et al., 2005; Khoo et al., 2006; Luo et al., 2013; Purohit et al., 2013). Subsequent to the design of these inhibitor peptides, natively expressed inhibitory proteins known as CaMKIINs were discovered. These small proteins were identified through a yeast two-hybrid screen where the CaMKII catalytic domain was used as bait (Chang et al., 1998; Chang et al., 2001), and while they have only been natively detected in the brain, they have been used effectively to target CaMKII activity. Notably, this peptide inhibitor binds with the kinase domain in the active conformation. Indeed, the CaMKIIN inhibitor has been used to limit myocyte death in response to MI, catecholamine stress, and ischemia–reperfusion (Swaminathan et al., 2011; Joiner and Koval, 2014) and reduce the occurrence of arrhythmogenic substrates (Koval et al., 2010) and in particular has utilized localization motifs to inhibit CaMKII within mitochondria or near plasma membrane domains (Joiner et al., 2012). Analysis of the peptide led to refinement and development of a 28-amino acid peptide inhibitor called CaMKIItide (Chang et al., 2001), while further refinement has led to the development of a shortened version only 19 amino acids long called CN19o (Coultrap and Bayer, 2011), with an IC50 of 0.4 nM. This was a >100-fold improvement in the IC50 than the native CaMKIIN, which also led to a stark improvement in the selectivity for CaMKII over other kinases (Coultrap and Bayer, 2011).

While the development and characterization of these highly selective peptide inhibitors has advanced cardiac research, there remains the rather significant challenge of delivery for therapeutic use. The greatest of these challenges is perhaps the limited bioavailability of short peptide sequences *in vivo*. Oral administration would provide limited enteral resorption and intravenous injection for the treatment of a chronic condition would offer challenges with continued compliance, let alone the quick half-life of a short peptide sequence in circulation (Otvos and Wade, 2014). Alternatively, gene therapy could be used to deliver a viral vector which expresses the short peptide. In the last 30 years, there have been four randomized clinical trials employing viral gene delivery for the treatment of HF with reduced ejection fraction (CUPID, CUPID-2, STOP-HF, and AC6) (Penny and Hammond, 2017). While the viral delivery was well tolerated in patients, the greatest burden they faced was insufficient gene transduction, where the patient with the lowest ejection fraction or those receiving the highest viral dosage were the only to experience improved endpoints. While these results do not preclude the future use of gene therapy, they do establish a more rigorous hurdle to overcome to pursue CaMKII inhibition.

A more novel and potentially robust opportunity, however, may reside in recent advances in nanotechnology. A recent report showed the successful delivery of peptide cargo to the heart through the use of biocompatible and biodegradable calcium phosphate nanoparticles introduced by inhalation (Miragoli et al., 2018). This strategy took advantage of the principle that oxygenated blood from the lungs moves from the pulmonary circulation to the heart first. This principle was supported by observations that nanoparticles and particulates from air pollution have been implicated in cardiac dysfunction and arrhythmia (Mills et al., 2009). Not only does the design of these particles allow for delivery to the heart and cell permeation but also protects peptides from enzymatic digestion. This approach has been used for successful delivery of a peptide impacting L-type Ca^2+^ channel expression, resulting in restored cardiac function in a mouse model of diabetic cardiomyopathy (Miragoli et al., 2018). Moreover, peptides have been delivered in a porcine model as well, with minimal delivery to tissues other than the heart. This mode of delivery not only represents a noninvasive and practical means of delivering novel cardiac peptide therapeutics but can take advantage of an already customized potency and precision of CaMKII peptide inhibitors like CaMKIItide or its derivative CN19o.

## Use of RNA Interference (RNAi) to Target CaMKII

When it comes to therapeutic treatment of human disease, including cardiac disease, therapeutic intervention falls within two major classes of FDA-approved drugs, small molecules and proteins (Verdine and Walensky, 2007). As has already been discussed, small molecules typically must overcome the burden of target specificity and efficacy and require significant investments of time and resources to make them available for use. Alternatively, protein-based drugs, which frequently take the form of antibodies, display higher specificity, but limitations on size, stability, and deliverability to the right cellular compartments are frequent barriers to their application (Verdine and Walensky, 2007). An alternative approach to the targeting of specific genes implicated in disease may be found in RNA therapeutics (**Figure 1C**). For close to 30 years, RNA drugs [short hairpin RNAs (shRNAs), small-interfering RNAs (siRNAs), microRNAs (miRNAs)] have been used at the bench to target protein expression to identify critical gene targets in disease and signaling pathways. However, their use in clinical settings has been a non-factor given that short single- and double-stranded RNA is extremely susceptible to nuclease digestion, may lead to immune system activation, and is too large and negatively charged to cross cell membranes (Crooke, 1999; Laina et al., 2018). However, significant advances in the design of RNA therapeutics have been able to overcome these challenges to the point that numerous clinical trials are currently underway employing RNAi-based drugs for both cardiac and noncardiac targets (Kaczmarek et al., 2017; Laina et al., 2018).

RNAi therapeutics fall into several different categories, including single-stranded antisense oligonucleotides (ASOs), double-stranded siRNAs, and miRNAs (Laina et al., 2018). ASOs are short (typically 20 base pairs in length) single-stranded synthetic molecules that take advantage of Watson–Crick base-pairing to bind a target mRNA to induce its degradation through endogenous RNAse activity (Crooke, 1999). Depending on the design of the ASO, it can also block ribosomal attachment to reduce target protein expression or even redirect splicing factors to lead to the inclusion or exclusion of targeted exons (Dominski and Kole, 1993; Havens and Hastings, 2016). The latter mode is the mechanism of action of the FDA-approved RNA therapeutic eteplirsen (Exondys 51, Sarepta Therapeutics) for treating Duchenne muscular dystrophy, which induces the skipping of exon 51 of the mutant dystrophin gene and restoring the proper translational reading frame for dystrophin expression (Dystrophy et al., 2013). Therapeutic siRNAs are also synthetic molecules used to silence target genes but are instead double-stranded molecules ranging in length from 19 to 25 base pairs (Siomi and Siomi, 2009). These molecules are recognized by Argonaute 2 and the RNA-induced silencing complex (RISC) and unwound into single-stranded components. The sense strand is degraded and the antisense binds to a target mRNA sequence to induce cleavage by Argonaute 2 and degradation by exonucleases (Sledz et al., 2003; Liu et al., 2004; Rand et al., 2005; Meister, 2013; Ozcan et al., 2015). Finally, miRNAs are endogenous small noncoding RNAs that target multiple mRNAs, silencing target gene expression either by mRNA degradation or translational inhibition. Typically, miRNAs target the 3'-untranslated region (UTR) of the target mRNA through a primary seed sequence eight nucleotides long, with the degree of repression being modulated by the complementarity and nucleotide composition of flanking sequences. These molecules are natively expressed within the cell either under regulation of their own discrete promoter or found within intronic sequences of coding genes. Therefore, therapeutic treatments have focused both on their inhibition through the delivery of antisense sequences acting as complementary decoys to the miRNA as well as direct overexpression of miRNA mimics.

In effect, each of these RNAi-based platforms could theoretically be applied to the inhibition of CaMKII in the heart. While an ASO could be designed with a particular target sequence in mind, there is also precedence for physiologic regulation of CaMKIIδ by a number of native miRNAs to abrogate cardiac remodeling (Wang et al., 2010; Aurora et al., 2012; Cha et al., 2013; He et al., 2013). A multitude of preclinical and clinical trials have employed the use of chemically modified RNAi backbones and encapsulation in nanoparticles to improve pharmacokinetics, escape nuclease activity, enhance target mRNA binding, and minimize toxicity (Lucas and Dimmeler, 2016). Importantly, a major potential advantage in using RNA technology is that it offers the potential for increased precision. Small molecule drugs frequently suffer from impacting unintended secondary targets due to either conservation of enzymatic binding sites (like ATP-competitive sites) or impacting unintended pathways. However, all protein expression is derived from coding mRNA containing nucleic acid sequences significantly more unique to each individual gene, thereby providing a layer of intended target specificity difficult to match through pharmacologic drug design. Even more notably, this precision can even extend to specific isoform targeting of CaMKII. Not only could an siRNA molecule be designed that would specifically target transcripts encoding the CaMKIIδ isoform, circumventing the burden of CaMKII inhibition in the brain where CaMKIIα and β predominate, but may even offer the advantage of specific splice variant targeting of CaMKIIδ by focusing on specific exon–exon junctions unique to each variant. As previous literature has been able to identify, there are splice variant-specific effects of CaMKIIδ in cardiac disease and remodeling, revealing a potential protective role of CaMKIIδ_B_, in comparison to the other cardiac variant CaMKIIδ_C_ (Little et al., 2009; Peng et al., 2010; Lu et al., 2011a).

Moreover, as mentioned, ASO-based therapeutics are also capable of influencing the access of splicing factors to induce the inclusion or skipping of certain exons based on their design. Through such a process, it would be possible to induce a state of increased exon inclusion that favors the variant CaMKIIδ_B_, leading to its increased expression relative to CaMKIIδ_C_. The consequence of this shift in relative abundances can alter the stoichiometry within the holoenzyme structure, imparting the localization pattern of the isoform that dominates within the complex. Such manipulation of the relative composition has led to the increased nuclear localization of the CaMKIIδ_C_ isoform when the holoenzyme is composed of more CaMKIIδ_B_ (Srinivasan et al., 1994). Given that nuclear activity of CaMKIIδ_C_ has been shown to lead to prosurvival mechanisms within cardiomyocytes and reduced pathologic remodeling (Little et al., 2009; Peng et al., 2010; Lu et al., 2011b), the manipulation of CaMKIIδ at the splicing level through RNA-based mechanisms offers a theoretically plausible mechanism for improving HF treatment that might not even require targeting CaMKII inhibition.

One of the remaining challenges for applying RNA-based CaMKII therapy is selective targeting of heart. Current application of these RNA agents is typically through intravenous or subcutaneous injection, exposing the drugs to systemic circulation. While chemical modification to these drugs can prevent crossing of the blood–brain barrier, this is likely insufficient to prevent undesired consequences for the ubiquitously expressed CaMKIIδ. While this barrier is not unique to RNA therapeutics (also a consideration for small molecule inhibition), there are options that are unique to RNA therapeutics for potentially overcoming this limitation, including gene therapy which could utilize cardiac specific promoters (limited to miRNAs for polymerase compatibility) or local delivery/injection directly into cardiac tissue. There is also exciting opportunity to combine emerging nanoparticle therapy (discussed in previous section) to carry RNA-based drugs instead of peptide cargos through the same inhalation delivery to deliver directly to the heart.

## Therapeutic Targeting of the CaMKII Signaling Pathway

When considering CaMKII as a therapeutic target, it is important to acknowledge opportunities for manipulating the CaMKII signaling pathway without directly targeting the kinase itself. As discussed previously, CaMKII regulates a large number of intracellular substrates (ion channels, transcription factors, Ca^2+^ handling proteins) that may serve as attractive targets for electrical and hypertrophic remodeling. Perhaps the best known example of therapy aimed at a downstream target in the CaMKII pathway comes from ranolazine, an approved antianginal agent that inhibits late Na^+^ current among other actions (Rayner-Hartley and Sedlak, 2015). Late Na^+^ current, through the actions of the sodium Ca^2+^ exchanger, may impact intracellular Ca^2+^, contributing to further activation of CaMKII as well as driving the incidence of arrhythmogenic DADs. Ranolazine has been used successfully in rodents and large animals to block these changes and prevent the development of hypertrophy, HF, and arrhythmias (Rastogi et al., 2008; Figueredo et al., 2011; Glynn et al., 2015; Liang et al., 2016; Ellermann et al., 2018; Nie et al., 2019). However, results from clinical trials have been mixed with data supporting that ranolazine is safe with questionable efficacy in preventing AF recurrence (RAFAELLO trial), ventricular tachycardia (VT) or ventricular fibrillation (VF) following implantation of cardioverter defibrillator (RAID trial), or improving functional capacity in hypertrophic cardiomyopathy (RESTYLE-HCM trial) (De Ferrari et al., 2015; Bengel et al., 2017; Olivotto et al., 2018; Zareba et al., 2018).

RyR2 is another potential therapeutic target downstream of CaMKII signaling (Wehrens et al., 2004; Guo et al., 2006; Yang et al., 2007; Wehrens, 2011). Compounds referred to as rycals are small molecules that aim to stabilize RyR2 in its closed conformation through enhanced association with its binding partner calstabin, without blocking the channel or impairing normal Ca^2+^ signaling. JTV519 (K201) is one such compound that has been shown to improve Ca^2+^ leak and left ventricular function in a canine model of HF (Yano et al., 2003). However, translatability of JTV519 is somewhat limited by several off-target effects on other cardiac channels (Kaneko et al., 2009). S107 is another more selective rycal (Bellinger et al., 2008) with demonstrated ability to protect against ventricular arrhythmias in a mouse model with a RyR2 gain-of-function mutation (Lehnart et al., 2008) in addition to reversing the development of HF in a mouse model of constitutively phosphorylated RyR2 (Shan et al., 2010). Molecules such as these are being prepared for clinical testing and represent promising avenues for attenuating the impact of CaMKII activation.

Besides searching downstream of CaMKII for druggable targets, it is important to consider that the functional consequences of any kinase is determined by a delicate balance between the kinase and antagonism by a phosphatase. The majority of phosphatase activity in the heart is due to type-1 phosphatases (PP1) and type-2 phosphatases [PP2A, PP2B (calcineurin)] (El-Armouche and Eschenhagen, 2009), which collectively have been implicated in either dephosphorylating proteins targeted by CaMKII kinase activity or targeting CaMKII directly, removing its autonomous activation (Chiang et al., 2016; Lubbers and Mohler, 2016) (**Figure 1D**). Therefore, it may be possible to balance increased CaMKII activity in disease by simultaneously activating associated phosphatases. For example, the PP2A activator FTY720 has been shown to attenuate hypertrophic remodeling and protect hearts from ischemic injury in the mouse (Egom et al., 2010; Liu et al., 2011; Liu et al., 2013). However, one possible complication with this approach is that increased phosphatase activity has been linked to development of HF (Parra and Rothermel, 2017). Transgenic activation of CaMKII has itself been shown to increase phosphatase activity (Zhang et al., 2002), but it is unclear if this is a compensatory change or a cofactor in driving disease. While maximally activated phosphatase activity is likely to amplify adverse remodeling as the transgenic models have shown, it may be possible to implement a more nuanced approach with the goal of maintaining proper balance between kinase and phosphatase activity. Given that the majority of phosphatase activity is supplied by just three genes, unlike kinases which have numerous members with highly specific functions, phosphatase targeting is regulated by a multitude of binding partners and anchoring proteins (Shi, 2009). Ca_v_1.2 dephosphorylation for example is dependent on PP2A and its interaction with regulatory subunits B56α and PR59 (Hall et al., 2006; Xu et al., 2010), while spinophilin and regulatory subunit PR130 target PP1 and PP2A to RyR2 (Bers, 2002; Lanner et al., 2010). Future studies may reveal critical subunits which direct phosphatases to relevant CaMKII targets, or to CaMKII itself, through which drug targeting or gene regulation would provide opportunity to influence the extent of that precise interaction. This may provide opportunity to limit adverse outcomes observed in global and unchecked phosphatase induction (Gergs et al., 2004; Hoehn et al., 2015), but clearly a great deal of work is still required to even establish proof of principle.

A unique opportunity for further discerning the potential impact of phosphatase activation may lie in the field of cancer therapeutics. Currently, cancer treatment strategies are beginning to consider phosphatase activators in earnest for tumor disruption (McClinch et al., 2018; Allen-Petersen and Sears, 2019; Remmerie and Janssens, 2019). It is well established that cardiac toxicity is a significant side effect following the use of the common cancer drug, doxorubicin, which has even been associated with CaMKII signaling (Yeh and Bickford, 2009; Sag et al., 2011). Therefore, it would be interesting to observe potential differences in the development of cardiomyopathy with the inclusion of these phosphatase activators when used in conjunction with doxorubicin to provide therapeutic insight and predictive data to the prospect of similar phosphatase activators being utilized for cardioprotective therapies.

Finally, when searching for alternative targets in the CaMKII pathway, it is worth considering scaffolding, cytoskeletal and other interacting proteins important for subcellular localization of CaMKII to specific signaling nanodomains. For example, a role for the cytoskeletal protein β_IV_-spectrin has been identified in targeting CaMKII to the cardiomyocyte intercalated disc to facilitate regulation of Na_v_1.5 (Hund et al., 2010). Genetic disruption of β_IV_-spectrin/CaMKII interaction selectively disrupted the subpopulation of CaMKII at the intercalated disc and prevented stress-induced phosphorylation of Na_v_1.5. Subsequent studies found that disruption of β_IV_-spectrin/CaMKII interaction abrogated maladaptive cardiac remodeling in a mouse model of pressure overload (Unudurthi et al., 2018). Thus, it may prove beneficial to develop pharmacological or genetic ways to interfere with spectrin/CaMKII interaction allowing for selective inhibition of a pathological CaMKII subpopulation while leaving the rest of the signaling pathway intact.

## Conclusion

Given the effectiveness of CaMKII inhibition in improving cardiac function and reducing arrhythmia burden in animal models of disease, it is reasonable to remain optimistic that targeting of CaMKII signaling has a future in cardiac translational therapy. While conventional delivery routes and strategies merit further investigation, it will be important going forward to be aggressive in the search for new delivery routes and targets that may accelerate translation of basic science discoveries to human patients. Recent advances in genetic techniques and/or particle deliveries provide entirely new paradigms for not just treating familial diseases but acquired forms as well. Therefore, the development of CaMKII therapeutics might not only represent a breakthrough for cardiac treatment but, depending on the routes taken, may open up an entirely new branch in the models we use to treat a broader range of disease.

## Author Contributions

DN, DG, and TH drafted and revised the manuscript.

## Funding

This work was supported by the National Institutes of Health (grant numbers R01-HL135096 and R01-HL134824 to TH) and the American Heart Association (postdoctoral fellowship to DN).

## Conflict of Interest

The authors declare that the research was conducted in the absence of any commercial or financial relationships that could be construed as a potential conflict of interest.
